# Induction chemotherapy followed by concurrent standard radiotherapy and daily low-dose cisplatin in locally advanced non-small-cell lung cancer

**DOI:** 10.1038/sj.bjc.6990693

**Published:** 1999-09

**Authors:** A Ardizzoni, F Grossi, T Scolaro, S Giudici, F Foppiano, L Boni, L Tixi, M Cosso, C Mereu, G Battista Ratto, V Vitale, R Rosso

**Affiliations:** Division of Medical Oncology I, Division of Radiation Oncology, Unit of Clinical Epidemiology and Trials, Service of Radiology, Istituto Nazionale per la Ricerca sul Cancro and Centro di Biotecnologie Avanzate, Largo R Benzi 10, Genova, 16132, Italy; Center of Respiratory Endoscopy, Chair of Thoracic Surgery, Università degli studi di Genova, Genova, Italy

**Keywords:** induction chemotherapy, concurrent chemoradiation in NSCLC, phase II study

## Abstract

Both induction chemotherapy and concurrent low-dose cisplatin have been shown to improve results of thoracic irradiation in the treatment of locally advanced non-small-cell lung cancer (NSCLC). This phase II study was designed to investigate activity and feasibility of a novel chemoradiation regimen consisting of induction chemotherapy followed by standard radiotherapy and concurrent daily low-dose cisplatin. Previously untreated patients with histologically/cytologically proven unresectable stage IIIA/B NSCLC were eligible. Induction chemotherapy consisted of vinblastine 5 mg m^−2^ intravenously (i.v.) on days 1, 8, 15, 22 and 29, and cisplatin 100 mg m^−2^ i.v. on days 1 and 22 followed by continuous radiotherapy (60 Gy in 30 fractions) given concurrently with daily cisplatin at a dose of 5 mg m^−2^ i.v. Thirty-two patients were enrolled. Major toxicity during induction chemotherapy was haematological: grade III–IV leukopenia was observed in 31% and grade II anaemia in 16% of the patients. The most common severe toxicity during concurrent chemoradiation consisted of grade III leukopenia (21% of the patients); grade III oesophagitis occurred in only two patients and pulmonary toxicity in one patient who died of this complication. Eighteen of 32 patients (56%, 95% CI 38–73%) had a major response (11 partial response, seven complete response). With a median follow-up of 38.4 months, the median survival was 12.5 months and the actuarial survival rates at 1, 2 and 3 years were 52%, 26% and 19% respectively. The median event-free survival was 8.3 months with a probability of 40%, 23% and 20% at 1, 2 and 3 years respectively. Induction chemotherapy followed by concurrent daily low-dose cisplatin and thoracic irradiation, in patients with locally advanced NSCLC, is active and feasible with minimal non-haematological toxicity. Long-term survival results are promising and appear to be similar to those of more toxic chemoradiation regimens, warranting further testing of this novel chemoradiation strategy. © 1999 Cancer Research Campaign


*British Journal of Cancer* (1999) **81**(2), 310–315
© 1999 Cancer Research Campaign
Article no. bjoc.1999.0693

### Induction chemotherapy followed by concurrent standard radiotherapy and daily low-dose cisplatin in locally advanced non-small-cell lung cancer

**A Ardizzoni[1], F Grossi[1], T Scolaro[2], S Giudici[2], F Foppiano[2], L Boni[3], L Tixi[1], M Cosso[4], C Mereu[5], G Battista Ratto[6], V Vitale[2] and R Rosso[1]**

[1]Division of Medical Oncology I, [2]Division of Radiation Oncology, [3]Unit of Clinical Epidemiology and Trials, [4]Service of Radiology, Istituto Nazionale per la Ricerca sul Cancro, Largo R Benzi 10, and Centro di Biotecnologie Avanzate, Largo R Benzi 10, 16132 Genova, Italy; [5]Center of Respiratory Endoscopy, [6]Chair of Thoracic Surgery, Università degli studi di Genova, Genova, Italy

**Summary** Both induction chemotherapy and concurrent low-dose cisplatin have been shown to improve results of thoracic irradiation in the treatment of locally advanced non-small-cell lung cancer (NSCLC). This phase II study was designed to investigate activity and feasibility of a novel chemoradiation regimen consisting of induction chemotherapy followed by standard radiotherapy and concurrent daily low-dose cisplatin. Previously untreated patients with histologically/cytologically proven unresectable stage IIIA/B NSCLC were eligible. Induction chemotherapy consisted of vinblastine 5 mg $m^{-2}$ intravenously (i.v.) on days 1, 8, 15, 22 and 29, and cisplatin 100 mg $m^{-2}$ i.v. on days 1 and 22 followed by continuous radiotherapy (60 Gy in 30 fractions) given concurrently with daily cisplatin at a dose of 5 mg $m^{-2}$ i.v. Thirty-two patients were enrolled. Major toxicity during induction chemotherapy was haematological: grade III–IV leukopenia was observed in 31% and grade II anaemia in 16% of the patients. The most common severe toxicity during concurrent chemoradiation consisted of grade III leukopenia (21% of the patients); grade III oesophagitis occurred in only two patients and pulmonary toxicity in one patient who died of this complication. Eighteen of 32 patients (56%, 95% CI 38–73%) had a major response (11 partial response, seven complete response). With a median follow-up of 38.4 months, the median survival was 12.5 months and the actuarial survival rates at 1, 2 and 3 years were 52%, 26% and 19% respectively. The median event-free survival was 8.3 months with a probability of 40%, 23% and 20% at 1, 2 and 3 years respectively. Induction chemotherapy followed by concurrent daily low-dose cisplatin and thoracic irradiation, in patients with locally advanced NSCLC, is active and feasible with minimal non-haematological toxicity. Long-term survival results are promising and appear to be similar to those of more toxic chemoradiation regimens, warranting further testing of this novel chemoradiation strategy.

© 1999 Cancer Research Campaign

**Keywords:** induction chemotherapy; concurrent chemoradiation in NSCLC; phase II study

Unresectable stage III represents nearly one-third of all NSCLC cases. Until a few years ago, standard treatment consisted of once daily fractionation thoracic high-dose radiotherapy. With this form of treatment, median survival was less than 1 year and 5-year survival rate nearly 5%, due to frequent systemic and local failures (Perez et al, 1987). However, more recent evidence has accumulated indicating that chemotherapy added to radiotherapy can increase long-term survival (Non-small Cell Lung Cancer Collaborative Group, 1995), and randomized studies comparing sequential chemoradiation to radiotherapy alone have consistently reported a better outcome in favour of the combined modality treatment. Among these studies, the Cancer and Leukemia Group B (CALGB), which has compared two courses of cisplatin–vinblastine induction chemotherapy followed by standard thoracic irradiation to irradiation alone, has reported a nearly 10% difference in actuarial survival in favour of combined modality which was maintained through 7 years of follow-up (Dillman et al, 1996). This result has been corroborated by a confirmatory study conducted by the Radiation Therapy Oncology Group (RTOG) and the Eastern Cooperative Oncology Group (ECOG) (Sause et al, 1995).

An alternative method of integrating chemotherapy and radiotherapy is to deliver these two treatment modalities concurrently, in order to take also advantage of possible radiosensitizing properties of some chemotherapeutic agents. Among these agents, cisplatin has been the most extensively used, due to a laboratory evidence of strong radiopotentiating activity (Dewitt, 1987). A series of randomized studies have assessed the role of low-dose cisplatin combined with radiotherapy, as compared to radiotherapy alone. One of these studies, which was conducted by the European Organisation for Research and Treatment of Cancer (EORTC), reported a statistically significant benefit for this type of concomitant chemoradiation treatment (Schaake-Koning et al, 1992). Interestingly, while the benefit of chemotherapy used as an induction to radiotherapy can be mainly attributed to an effect on systemic disease (Le Chevalier et al, 1991; Crinò et al, 1993), cisplatin used concurrently with radiotherapy appears to improve essentially local control (Soresi et al, 1988; Schaake-Koning et al, 1992). This observation provides the rationale for combining the two modalities of integrating chemotherapy and radiotherapy (induction chemotherapy followed by concurrent chemoradiation) aimed at associating the benefits of an improved control on loco-regional disease and on micrometastases. The present study was

*Received 27 October 1998*
*Revised 25 March 1999*
*Accepted 12 April 1999*

*Correspondence to:* A Ardizzoni designed to test this hypothesis and to verify the activity and feasibility of a novel chemoradiation programme, derived from a combination of CALGB (Dillman et al, 1996) and EORTC (Schaake-Koning et al, 1992) study best arms, consisting of induction chemotherapy followed by standard radiotherapy and simultaneous daily low-dose cisplatin.

### PATIENTS AND METHODS

### Eligibility

Eligibility criteria for this trial included a cytological or histological diagnosis of NSCLC, clinical stage IIIA/B disease judged unresectable by a thoracic surgeon (GBR) and presence of measurable or evaluable disease according to World Health Organization (WHO) criteria. Patients were required to have a WHO performance status equal or less than 2, to be no more than 70 years old and to have a weight loss less than 10% of total body weight in the preceding 6 months. At the time of study entry, eligible patients were required to have haemoglobin greater than 10 g $dl^{-1}$, WBC count higher than 4000 $\mu l^{-1}$, a platelet count higher than 100 000 $\mu l^{-1}$, bilirubin less than 2.5 mg $dl^{-1}$ and creatinine less than 1.5 mg $dl^{-1}$. Eligibility for this trial included also the presence of a forced expiratory volume in 1 s ($FEV_1$) higher than 1 l. No previous chemotherapy or radiotherapy was allowed.

Patients were excluded from study participation if they had any of the following conditions: malignant pleural effusion or involvement of supraclavicular lymph nodes, any concomitant serious illness, infectious disease in the previous 3 weeks, life-expectancy less than 6 months, stage I–II disease, superior vena cava syndrome, post-obstructive pneumonia, life-threatening haemoptysis, small-cell lung cancer component in the biopsy specimen, myocardial infarction in the previous 6 months, clinical evidence of cardiac failure and/or uncontrolled arrhythmia. Patients with previous or current malignancies at other sites, with the exception of in situ carcinoma of the cervix uteri and/or basal cell carcinoma of the skin were also excluded.

Pretreatment evaluation included a complete medical history and physical examination with neurologic evaluation, chest X-ray and computerized tomography (CT) scan, abdominal CT scan or ultrasonography, fiberoptic bronchoscopy, pulmonary function tests, audiograms, blood chemistry including complete blood cell counts, electrolytes, blood urea nitrogen, serum creatinine, liver function tests, serum tumour markers (CEA, CYFRA, NSE), urinalysis and ECG.

### Treatment

The entire treatment was delivered on an outpatient basis. Induction chemotherapy included vinblastine 5 mg $m^{-2}$ given as an intravenous (i.v.) bolus on days 1, 8, 15, 22 and 29, and cisplatin 100 mg $m^{-2}$ given i.v. over a 30- to 60-min period on days 1 and 22 along with 2 litres NS hydration and forced diuresis.

Continuous thoracic irradiation with concurrent cisplatin started from day 43. Cisplatin was administered bolus at a 5 mg $m^{-2}$ daily dose, 1–2 h before each radiotherapy administration. Patients were required to have an oral intake of at least 2 l of fluids daily. Radiotherapy was delivered with high energy photon beams from a 15–18 MeV linear accelerator. The total dose delivered was 60 Gy in 30 fractions, 5 fractions per week (Monday to Friday). Target volume included the prechemotherapy primary tumour with 2-cm margins, ipsilateral hilum and mediastinum from the sternal notch to 5 cm below the carina. Ipsilateral supraclavicular nodes were included for upper lobe primary tumours or when high mediastinal lymph nodes (stations 1 and 2) were involved. Inferior mediastinal nodes were treated in case of lower lobe tumours. When left upper lobe was the site of the neoplasm, contralateral supraclavicular nodes were included in the target volume. Two opposite antero-posterior fields were employed for the first 22–24 fractions.

After the first 44–48 Gy were delivered, the field was reduced to include only the primary tumour and gross lymph node volume with 1-cm margin for an additional 12–16 Gy. This latter treatment was realized with 2–3 computer planned fields avoiding the spinal cord (where the maximum dose should not exceed 48 Gy). All patients were submitted to CT simulation and volumes were drawn on CT images. Customized blocks were defined with beams-eye-view support. Dose distribution calculation was performed employing several CT images using 2D multiple slices treatment planning system. Normalization and weighing points were identified with isocentre dose and homogeneity according to ICRU recommendation (International Commission on Radiation Units and Measurements, 1993).

### Dose modifications

The use of haemopoietic growth factors was not allowed. Dose modifications were planned according to toxicity. Cisplatin dose, during induction chemotherapy, had to be reduced of 25% if the preceding course had produced grade IV leukopenia and/or thrombocytopenia. If myelosuppression precluded the starting of the second chemotherapy cycle on day 22, the treatment was postponed for 1 week or to complete resolution of toxicity. Vinblastine dosing was omitted in case of grade III–IV haematological toxicity. Chemoradiation could be interrupted for periods up to 10 treatment days for grade ≥ III oesophageal toxicity and for grade IV haematological toxicity.

### Follow-up

Blood count and serum creatinine were obtained weekly during treatment. Following completion of radiation therapy, patients were seen at 4-week intervals for 6 months, then at 8-week intervals for 6 months, then every 3 months for 2 years and every 6 months thereafter. Follow-up evaluation included: a complete medical history and physical examination with neurologic evaluation, blood chemistry including complete blood cell counts, electrolytes, blood urea nitrogen, serum creatinine, liver function tests, serum tumour markers (CEA, CYFRA, NSE), urinalysis, chest CT scan and abdominal CT scan or ultrasonography at 3 and 6 months after completion of treatment and every 6 months thereafter. A fiberoptic bronchoscopy was obtained within 3 months from the end of treatment in responding patients and every year thereafter. Pulmonary function tests and audiograms were repeated only once after completion of treatment. Patients have been evaluated for tumour response based on CT scan at 2–3 months from radiotherapy termination. More sophisticated work-up was performed only if indicated.

Complete response (CR) was defined as complete disappearance of all signs of disease on CT or the presence of minimal CT abnormalities attributable to radiation fibrosis which did not change in subsequent CT scans. Definition of CR required a

© 1999 Cancer Research Campaign 

**Table 1** Patient characteristics

| Characteristic | No. of patients (%) (*n* = 32) |
|---|---|
| Male/female | 31/1 |
| Age (years) | |
| Range | 44–70 |
| Median | 60.5 |
| PS | |
| 0 | 17 (53) |
| 1 | 15 (47) |
| Cell type | |
| Adenocarcinoma | 10 (31) |
| Squamous cell | 22 (69) |
| Clinical Stage | |
| IIIA | 10 (37) |
| T1–2N2 | 4 |
| T3N2 | 6 |
| IIIB | 22 (63) |
| T1–3N3 | 7 |
| T4N0–2 | 13 |
| T4N3 | 2 |

negative fiberoptic bronchoscopy with biopsy. A partial response (PR) was a reduction of 50% or more in the sum of product of the longest perpendicular diameters of the tumour, measured with CT scan carried out at 2–3 months from the end of treatment. Stable disease (SD) was indicated by a less than 50% reduction or less than 25% increase in tumour size. Early death was defined as a death occurring before response evaluation. All CT scans were reviewed by a committee including one radiologist (MC), two oncologists (AA, FG), one radiotherapist (TS) and one chest physician (CM). All members of the committee had to agree about the judgment on response. Patients who progressed after radiotherapy were offered a second-line treatment or best supportive care only. No surgery was planned after the chemoradiation programme. Chemotherapy and chemoradiation toxicities were evaluated according to WHO criteria (WHO, 1979).

### Study design and statistical analysis

This study was designed as a prospective, single-institution, non-randomized phase II trial. The trial was conducted under the auspices of the EORTC Lung Cancer Cooperative Group who significantly contributed to the design of the study.

The primary aim of the study was to assess the activity and the toxicity of induction chemotherapy followed by radiotherapy combined with daily cisplatin in unresectable stage III NSCLC patients who had not received any prior chemo- and/or radiotherapy. The main end point was response rate.

To calculate the sample size we adopted Simon's minmax two-stage design for phase II clinical trials which minimizes the expected number of patients to be accrued if a combination has low activity (Simon, 1989).

At the time of the study planning, a regimen with a response rate of 50% in this population of patients was considered worthy of further study. The sample size was calculated on the following assumptions: alpha and beta errors were both set at 10% while P0 and P1, defined according to Simon (1989), were set at 30% and 50% respectively. In the first stage, 28 patients had to be enrolled. If ≤ 7 responses were observed, the accrual had to be stopped and the treatment protocol rejected. In the case of > 7 responses,

**Table 2** Maximum tumour response after the end of treatment

| Response | Number of patients (%) (*n* = 32) |
|---|---|
| Complete response | 7 (22%) |
| Partial response | 11 (34%) |
| Stable disease | 1 (3%) |
| Early death | 5 (16%) |
| Disease progression | 7 (22%) |
| Dropouts | 1 (3%) |

11 more patients had to be accrued. The treatment regimen had to be accepted if ≥ 16 responses out of 39 evaluable patients were observed.

All accrued patients were included in the final analysis of response rate and survival on an 'intention to treat' principle, thereby including also early deaths, early progressions and protocol violations. Overall survival (OS) was computed as the time from start of treatment to death or last visit. Event-free survival (EFS) was computed as the time from start of treatment to relapse at any site, death, or last visit. Estimates of OS and EFS were calculated according to the Kaplan–Meier product-limit method (Peto et al, 1977). Follow-up times were truncated on 28 February, 1998. The protocol was approved by the local Ethical Committee and Institutional Review Board. An informed consent was obtained from all registered patients.

### RESULTS

### Patient characteristics

A total of 32 patients were accrued into the study from June 1993 to July 1997. The accrual was terminated earlier, as soon as we reached the number of objective responses required by the statistical design to consider the regimen worthy of further testing. All patients, except one, were males; the median age was 60.5 (range 44–70 years). Seventeen patients (53%) had WHO performance status (PS) 0, 15 patients (47%) had PS 1. Histology consisted of adenocarcinoma in ten (31%) patients and squamous cell carcinoma in 22 (69%) patients. Ten (31%) patients had clinical stage IIIA disease and 22 (69%) had stage IIIB disease. The characteristics of the 32 patients are listed in Table 1.

### Activity

As shown in Table 2, 18 of 32 patients (56%, 95% confidence interval (CI) 38–73%) had a major response (11 PR, seven CR). Five patients died before response evaluation (three of early progression, one of pulmonary toxicity and one of cardiac infarction); seven patients were classified as progressive and one as stable at the time of response assessment. One patient was excluded from analysis because he underwent resection before completion of chemoradiation.

Most relapses or progression occurred locally. The first sites of initial disease progression are listed in Table 3. As of 28 February 1998, six of seven patients with CR and one of 11 patients with PR remain alive with no recurrence or progression of disease at 7, 27, 37, 38, 49, 54 and 56 months respectively.

 © 1999 Cancer Research Campaign

**Table 3** Sites of initial disease progression

| Variable | Number of patients (%) (*n* = 20) |
|---|---|
| Distant alone | 4 (20) |
| Local alone | 14 (70) |
| Distant+local | 1 (5) |
| Unknown | 1 (5) |

**Table 4** Chemotherapy and chemoradiation toxicity

| Toxicity | Grade | Chemotherapy No. of patients (%) (*n* = 32) | Chemoradiation No. of patients (%) (*n* = 28) |
|---|---|---|---|
| Anaemia | I | 8 (25) | 4 (14.3) |
| | II | 5 (15.6) | 6 (21.4) |
| | III | 0 | 2 (7.1) |
| Leukopenia | I | 6 (18.7) | 7 (25) |
| | II | 13 (40.6) | 7 (25) |
| | III | 5 (15.6) | 6 (21.4) |
| | IV | 5 (15.6) | 0 |
| Thrombocytopenia | I | 2 (6.2) | 2 (7.1) |
| | II | 1 (3.1) | 3 (10.7) |
| | III | 0 | 2 (7.1) |
| | IV | 1 (3.1) | 0 |
| Stomatitis | I | 2 (6.2) | 19 (67.8) |
| Oesophagitis | II | 2 (6.2) | 1 (3.5) |
| | III | 1 (3.1) | 2 (7.1) |
| Nausea/vomiting | I | 12 (37.5) | 10 (35.7) |
| | II | 1 (3.1) | 0 |
| Asthenia | I | 9 (28.1) | 2 (7.1) |
| Paraesthesias | I | 5 (15.6) | 3 (10.7) |
| Tinnitus | II | 3 (9.3) | 1 (3.5) |
| Creatinine | I | 4 (12.5) | 2 (7.1) |
| Pulmonary toxicity | I | 0 | 1 (3.5) |

### Toxicity

Thirty-two patients were evaluable for induction chemotherapy toxicity. Grade III–IV leukopenia was observed in 10/32 (31%) cases, grade II anaemia in 5/32 (16%) and grade IV thrombocytopenia in only one case (3%). Non-haematological toxicity consisted mainly of grade I paraesthesia which occurred in 5/32 patients (16%).

Twenty-eight patients were evaluable for chemoradiotherapy plus daily cisplatin toxicity. Four patients could not be evaluated for radiotherapy toxicity because of early progressive disease or death before starting irradiation. Haematological toxicity consisted of grade III leukopenia observed in 6/28 patients (21%), grade III anaemia in 2/28 (7%) and grade III thrombocytopenia in 2/28 (7%) patients respectively. Oesophagitis, although frequent (22/28), was severe in only two patients. Only one case of radiation pneumonitis was observed. No lung function tests impairment was detected at completion of treatment. Four cases of tinnitus were seen. No objective neurological deficit, attributable to peripheral neurotoxicity, could be documented. No cumulative > grade I renal toxicity was observed. One treatment-related death due to respiratory failure was observed in the only patient who developed radiation pneumonitis (Table 4).

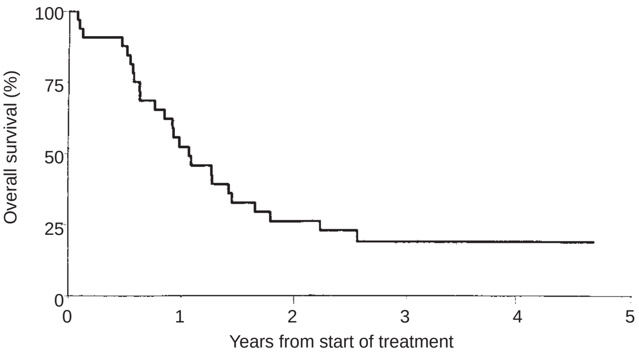

**Figure 1** Actuarial Kaplan–Meier survival curve of entire patient population

### Chemotherapy and chemoradiotherapy delivered

Chemoradiation, including daily cisplatin and thoracic irradiation, was completed as planned by the protocol in all patients who began the treatment. Some patients had brief delays (less than 1 week) due to toxicity or technical reasons. Mean projected/ planned dose % of cisplatin and vinblastine during induction chemotherapy were 95% and 85% respectively. The main reason for reduced dose intensity of vinblastine was the omission of day 8 or 15 dosing, due to haematological toxicity. During induction chemotherapy, there was a treatment delay in ten patients, a cisplatin dose reduction in two and a vinblastine dose reduction in 17 due to toxicity, primarily myelosuppression.

### Survival

All 32 patients enrolled into the trial were included in the survival analyses. The median follow-up was 38.4 (range 7.5–56) months. Twenty-five patients have died. Seven patients were surviving without evidence of recurrence or progression at the last follow-up and six of them were disease-free after a minimum follow-up of 2 years. The median overall survival was 12.5 months. The actuarial survival at 1, 2 and 3 years was 52%, 26% and 19% respectively (Figure 1). The median event free survival was 8.3 months with a probability of 40%, 23% and 20% at 1, 2 and 3 years respectively.

### DISCUSSION

Currently, the combination of chemotherapy and radiotherapy represents the standard treatment of locally advanced unresectable NSCLC (American Society of Clinical Oncology, 1997). However, the best timing of combining the two treatment modalities remains uncertain. The sequential approach, with chemotherapy preceding irradiation, has been the most extensively used in the trials performed to demonstrate superiority of combined modality treatment over radiotherapy alone. In addition, it has little additive toxicity and no technical problems. Improved results of sequential chemo-irradiation over radiotherapy alone are supposed to derive mainly from the effect of induction chemotherapy on micrometastatic disease (Le Chevalier et al, 1991; Crinò et al, 1993). As to the concurrent use of chemotherapy and irradiation, although the number of randomized studies in which this modality has been compared to radiotherapy alone is small, most of these trials have also been positive (Jeremic et al, 1996), including those where

© 1999 Cancer Research Campaign chemotherapy consisted of single agent cisplatin (Schake-Koning et al, 1992). This type of combined modality treatment appears to exert its action mainly by improving local control, probably as a result of a radiopotentiating effect of chemotherapy. However, with the exception of those regimens where concurrent chemotherapy consisted of single-agent cisplatin, most regimens using combination chemotherapy or third-generation agents concurrently with thoracic irradiation have shown severe additive toxicity, particularly oesophagitis and pneumonitis (Lee et al, 1996; Reckzeh et al, 1996; Frasci et al, 1997). Recently, there have been a number of attempts to further improve the results of combined chemoradiation by using more recently developed chemotherapy regimens and by adding sequential chemotherapy to concurrent chemoradiation in order to take possible advantage of two different chemoradiation strategies combined together (Greco et al, 1996; Choy et al, 1997; Isokangas et al, 1998). Although preliminary results have been extremely promising in terms of activity, the enhanced normal-tissue toxicity resulting from these novel chemoradiation regimens is worrying.

In the present study, we have attempted to combine sequential and concurrent chemoradiation without incurring in prohibitive toxicities, by integrating two well known regimens, both proved highly feasible and superior to radiotherapy alone. The sequential part of our regimen was that of the CALGB study number 8433 (Dillman et al, 1996), while the concurrent part was derived from the EORTC study number 08844 (Schaake-Koning et al, 1992), with the exception that radiotherapy was continuous as opposed to split-course, and, consequently, the dose of cisplatin reduced, as previously piloted by the Southwest Oncology Group (SWOG) (Hazuka et al, 1994).

The activity of our regimen seems promising. The accrual was terminated earlier as we met the end point of the study (16 objective responses). Median survival was 12.5 months and 1-, 2- and 3-year actuarial survival rate were 52%, 26% and 19% respectively. Although this was a phase II study with a relatively small number of patients accrued in a long period of time, our survival data compare favourably with those of CALGB and EORTC trials. In these studies, corresponding figures were 54%, 26% and 24% survival at 1, 2 and 3 years respectively in the CALGB study and 54%, 26% and 16% in the EORTC study.

In view of the lower toxicity of our chemoradiation programme, the results obtained in this study compare favourably also with those of more recent combined modality regimens. In fact, oesophagitis, although common, was severe requiring tube feeding in only two cases. In contrast, incidence of severe oesophagitis in most recently developed concurrent chemoradiation programmes was reported as high as 45% (Greco et al, 1996). In addition, radiation pneumonitis, which has been reported as one of the most serious complications with the concurrent use of chemotherapy and radiation (Reckzeh et al, 1996) was almost absent in our study. Most of our patients underwent serial pulmonary function tests to assess possible subclinical pulmonary damage. Significant changes of these tests have never been observed. At the beginning of our study, we were concerned of possible cumulative neurological and auditory cisplatin toxicity. In fact, the projected cumulative cisplatin dose of our regimen was 350 mg $m^{-2}$. For this reason, serial audiometric tests and clinical neurological examinations were planned. No objective loss of neurologic or auditory function was observed in any of our patients.

The most important toxicity of our regimen was myelosuppression during induction chemotherapy. The combination of cisplatin and vinblastine was chosen as induction chemotherapy for our programme in order to be consistent with the CALGB regimen from which our chemoradiation programme was derived. However, the use of this regimen is no longer justified at the present time given the availability of less toxic and more active last generation chemotherapy regimens (Giaccone et al, 1998).

In conclusion, we believe that our chemoradiation programme, as described in this report, is promising in terms of anti-tumour activity and long-term survival, highly feasible in terms of practicality and devoid of significant additive pulmonary and oesophageal toxicity and of cumulative cisplatin toxicity. Therefore, further studies with this combined modality approach are justified. Exploring the possible superiority of sequential plus concurrent chemoradiation over either single combined modality treatment is, in our opinion, one of the research priorities in the field of multimodality therapy of locally advanced NSCLC. In this respect, our regimen can be considered as an alternative to more toxic and expensive integrated regimens.

Despite the effort to improve local control by adding chemotherapy to radical radiotherapy in a concurrent and sequential fashion, thoracic progression still represents a major reason of failure. New strategies to further improve local control, such as the addition of post-treatment radical surgery (Eberhardt et al, 1998) or the use of hyperfractionated radiotherapy (Choy et al, 1997; Frasci et al, 1997) should also be pursued.

### ACKNOWLEDGEMENTS

Supported by AIRC 1995–1996 grant. The authors are indebted to N van Zandwijk, A Gregor and G Giaccone for their helpful suggestions in designing the present study.

### REFERENCES

American Society of Clinical Oncology (1997) Practice guidelines for the treatment of unresectable non-small-cell lung cancer. *J Clin Oncol* **15**: 2996–3018

Choy H, DeVore RF 3rd, Hande KR, Porter LL, Rosenblatt P, Yunus F, Schlabach L, Smith C, Shyr Y, LaPorte K and Johnson DH (1997) Preliminary analysis of a phase II study of paclitaxel, carboplatin, and hyperfractionated radiation therapy for locally advanced inoperable non-small-cell lung cancer. *Semin Oncol* **24**: S12-21–S12-26

Crinò L, Latini P, Meacci M, Corgna E, Maranzano E, Darwish S, Minotti V, Santucci A and Tonato M (1993) Induction chemotherapy plus high-dose radiotherapy versus radiotherapy alone in locally advanced unresectable non-small-cell lung cancer. *Ann Oncol* **4**: 847–851

Dewitt L (1987) Combined treatment of radiation and cisdiamminedichloroplatinum (II): a review of experimental and clinical data. *Int J Radiat Oncol Biol Phys* **13**: 403–426

Dillman RO, Herndon J, Seagren SL, Eaton WL Jr and Green MR (1996) Improved survival in stage III non-small-cell lung cancer: seven-year follow-up of cancer and leukemia group B (CALGB) 8433 trial. *J Natl Cancer Inst* **88**: 1210–1215

Eberhardt W, Wilke H, Stamatis G, Stuschke M, Harstrick A, Menker H, Krause B, Müeller MR, Stahl M, Flasshove M, Budach V, Greschuchna D, Konietzko N, Sack H and Seeber S (1998) Preoperative chemotherapy followed by concurrent chemoradiation therapy based on hyperfractionated accelerated radiotherapy and definitive surgery in locally advanced non-small-cell lung cancer: mature results of a phase II trial. *J Clin Oncol* **16**: 622–634

Frasci G, Comella P, Scoppa G, Guida C, Gravina A, Fiore F, Casaretti R, Daponte A, Parziale A and Comella G (1997) Weekly paclitaxel and cisplatin with concurrent radiotherapy in locally advanced non-small-cell lung cancer: a phase I study. *J Clin Oncol* **15**: 1409–1417

Giaccone G, Splinter TA, Debruyne C, Kho GS, Lianes P, van Zandwijk N Pennucci MC, Scagliotti G, van Meerbeeck J, van Hoesel Q, Curran D, Sahmoud T and Postmus PE (1998) Randomized study of paclitaxel-cisplatin versus cisplatin-teniposide in patients with advanced non-small-cell lung cancer. *J Clin Oncol* **16**: 2133–2141

© 1999 Cancer Research Campaign

Greco FA, Stroup SL, Gray JR and Hainsworth JD (1996) Paclitaxel in combination chemotherapy with radiotherapy in patients with unresectable stage III non-small-cell lung cancer. *J Clin Oncol* **14**: 1642–1648

Hazuka MB, Crowley JJ, Bunn PA Jr, ORourke M, Braun TJ and Livingston RB (1994) Daily low-dose cisplatin plus concurrent high-dose thoracic irradiation in locally advanced unresectable non-small-cell lung cancer: results of a phase II Southwest Oncology Group study. *J Clin Oncol* **12**: 1814–1820

International Commission on Radiation Units and Measurements (1993) Prescribing, Recording, and Reporting Photon Beam Therapy (Report 50)

Isokangas OP, Joensuu H, Halme M, Jekunen A and Mattson K (1998) Paclitaxel (Taxol) and carboplatin followed by concomitant paclitaxel, cisplatin and radiotherapy for inoperable stage III NSCLC. *Lung Cancer* **20**: 127–133

Jeremic B, Shibamoto Y, Acimovic L and Milisavljevic S (1996) Hyperfractionated radiation therapy with or without concurrent low-dose daily carboplatin-etoposide for stage III non-small-cell lung cancer: a randomized study. *J Clin Oncol* **14**: 1065–1070

Le Chevalier T, Arriagada R, Quoix E, Ruffie P, Martin M, Tarayre M, Lacombe-Terrier MJ, Douillard JY and Laplanche A (1991) Radiotherapy alone versus combined chemotherapy and radiotherapy in nonresectable non-small-cell lung cancer: first analysis of a randomized trial in 353 patients. *J Natl Cancer Inst* **83**: 417–423

Lee JS, Scott C, Komaki R, Fossella FV, Dundas GS, McDonald S, Byhardt RW and Curran WJ Jr (1996) Concurrent chemoradiation therapy with oral etoposide and cisplatin for locally advanced inoperable non-small-cell lung cancer: RTOG protocol 91-06. *J Clin Oncol* **14**: 1055–1064

Non-small Cell Lung Cancer Collaborative Group (1995) Chemotherapy in non-small cell lung cancer: a meta-analysis using updated data on individual patients from 52 randomised trials. *BMJ* **311**: 899–909

Perez CA, Pajak TF, Rubin P, Simpson JR, Mohiuddin M, Brady LW, Perez-Tamayo R and Rotman M (1987) Long-term observations of the patterns of failure in patients with unresectable non-oat cell carcinoma of the lung treated with definitive radiotherapy. Report by the Radiation Therapy Oncology Group. *Cancer* **59**: 1874–1881

Peto R, Pike MC, Armitage P, Breslow NE, Cox DR, Howard SV, Mantel N, McPherson K, Peto J and Smith PG (1977) Design and analysis of randomized clinical trials requiring prolonged observation of each patient. II. Analysis and examples. *Br J Cancer* **35**: 1–39

Reckzeh B, Merte H, Pflüger KH, Pfab R, Wolf M and Havemann K (1996) Severe lymphocytopenia and interstitial pneumonia in patients treated with paclitaxel and simultaneous radiotherapy for non-small-cell lung cancer. *J Clin Oncol* **14**: 1071–1076

Sause WT, Scott C, Taylor S, Johnson D, Livingston R, Komaki R, Emami B, Curran WJ, Byhardt RW, Turrisi AT, Rashid Dar A and Cox JD (1995) Radiation therapy oncology group (RTOG) 88-08 and eastern cooperative oncology group (ECOG) 4588: preliminary results of a phase III trial in regionally advanced, unresectable non-small-cell lung cancer. *J Natl Cancer* **87**: 198–205

Schaake-Koning C, van den Bogaert W, Dalesio O, Festen J, Hoogenhout J, van Houtte P, Kirkpatrick A, Koolen M, Maat B, Nijs A, Renaud A, Rodrigus P, Schuster-Uitterhoeve L, Sculier J-P, van Zandwijk N and Bartelink H (1992) Effects of concomitant cisplatin and radiotherapy on inoperable non-small-cell lung cancer. *N Engl J Med* **326**: 524–530

Simon R (1989) Optimal two-stage design for phase II clinical trials. *Controlled Clin Trials* **10**: 1–10

Soresi E, Clerici M, Grilli R, Borghini U, Zucali R, Leoni M, Botturi M, Vergari C, Luporini G and Scoccia S (1988) A randomized clinical trial comparing radiation therapy vs radiation therapy plus cis-dichlorodiammine platinum (II) in the treatment of locally advanced non-small cell lung cancer. *Semin Oncol* **15**(suppl 7): 20–25

WHO (1979) *WHO Handbook for Reporting Results of Cancer Treatment*. World Health Organization: Geneva

*© 1999 Cancer Research Campaign*
